# A Compressed Sensing Based Method for Reducing the Sampling Time of A High Resolution Pressure Sensor Array System

**DOI:** 10.3390/s17081848

**Published:** 2017-08-10

**Authors:** Chenglu Sun, Wei Li, Wei Chen

**Affiliations:** 1 Center for Intelligent Medical Electronics, Department of Electronic Engineering, School of Information Science and Technology, Fudan University, Shanghai 200433, China; 16110720101@fudan.edu.cn (C.S.); 16210720032@fudan.edu.cn (W.L.); 2 Shanghai Key Laboratory of Medical Imaging Computing and Computer Assisted Intervention, Shanghai 200000, China

**Keywords:** noninvasive monitoring, respiratory rate monitoring, pressure distribution imaging, compressed sensing, pressure sensor array

## Abstract

For extracting the pressure distribution image and respiratory waveform unobtrusively and comfortably, we proposed a smart mat which utilized a flexible pressure sensor array, printed electrodes and novel soft seven-layer structure to monitor those physiological information. However, in order to obtain high-resolution pressure distribution and more accurate respiratory waveform, it needs more time to acquire the pressure signal of all the pressure sensors embedded in the smart mat. In order to reduce the sampling time while keeping the same resolution and accuracy, a novel method based on compressed sensing (CS) theory was proposed. By utilizing the CS based method, 40% of the sampling time can be decreased by means of acquiring nearly one-third of original sampling points. Then several experiments were carried out to validate the performance of the CS based method. While less than one-third of original sampling points were measured, the correlation degree coefficient between reconstructed respiratory waveform and original waveform can achieve 0.9078, and the accuracy of the respiratory rate (RR) extracted from the reconstructed respiratory waveform can reach 95.54%. The experimental results demonstrated that the novel method can fit the high resolution smart mat system and be a viable option for reducing the sampling time of the pressure sensor array.

## 1. Introduction

The rapid advances in the Internet of Things (IoT) technology, sensor technique and the conception of the unobtrusively monitoring offer opportunities to monitor biomedical signals by a variety of IoT assets, like garment, mobile devices and even living goods, for different groups of people ranging from neonates to elderly [1,2,3,4,5].

As examples of such systems, a watch-type vital-sign sensor [6] has been proposed to monitor body movement. Y. Peng presents multimodality sensors system for sleep monitoring based on audio and video signals [7]. In [8], investigators use an infrared camera in placement of the traditional camera to obtain the physiological signal which is invisible by naked eyes. An intelligent eye mask with user friendly interface was proposed to obtain the EOG signals for measuring sleep stage and quality [9]. A smart jacket used for monitoring the seizure for neonates was presented [10]. And the walking gait monitoring and real-time activity classification can be accomplished with an ear-worn sensor [11,12]. And some interactive wearable systems which utilized various sensors can be used for the stroke patients’ upper body rehabilitation or monitoring body movements of neonates [13,14]. It can be seen that those noninvasive and unrestrained sensing techniques open a novel way for physiological signal detection conveniently and comfortably.

Recently, many researchers have attempted to develop many kind of mats to detect the information such as body’s pressure distribution, respiration, and even heart rate. Existing work includes, for example, a noninvasive pneumatics-based system applied to measure heartbeat, respiration, snoring, and body movements of a subject in bed [15], an unobtrusive framework for sleep stage identification and measuring pressure distribution based on a pressure-sensitive e-textile bed sheet [16], an unobtrusive bed mattress system using pressure sensors to measure multi-channel respiration signals for detecting the sleep apnea-hypopnea syndrome [17], and a smart mat embedded with 64 pressure sensors to measure pressure distribution of a neonate inside an incubator [18]. Those unobtrusive ways to obtain the physiological signals and monitor the occurrence of some corresponding diseases can be applied in sleep monitoring, health assessment and MRI-compatible breathing gates etc. However, those previous work related to the mat has limitations like difficult to practical use, uncomfortableness, inconvenience, low resolution and weak anti-interference ability etc.

To solve the problems mentioned above, we developed a prototype of smart mat system with flexible sensor array [19], which employed soft materials such as the sensors and printed fabric electrodes. In order to generate high-resolution pressure distribution image and offer more image details, the prototype was embedded with 1024 pressure sensors in a 28.8 cm × 28.8 cm area. With the help of high-resolution pressure sensor array (HRPSA), the smart mat with high resolution pressure distribution will be used for analyzing sleep by extracting many important physiological features such as the respiration, sleep posture, body movements, body pressure distribution and so on. Thus the HRPSA mat system is expected to be a feasible option for unobtrusive sleep monitoring. At the same time, the HRPSA can also be applied to prevent ulcer for post-surgical patients and for on bed rehabilitation exercises. And the similar HRPSA might be used for making the sensitive skin for robots, accordingly, the different patterns of robot-object contacts, collisions and tactile human-robot interaction might be recognized [20,21] .

Although we had overcome some limitations mentioned before, we encountered new problems that it takes more time to acquire the signal of all the sensors due to the large quantity of sensors, and the time for generating one frame image was 0.76 s and the sampling frequency of the respiratory waveform is 1.315 Hz for that system. In this case, the pressure distribution image and the respiratory waveform are less resistant to interference, and easy to be distorted due to the longer sampling time for one frame.

Therefore we proposed to reduce the sampling time and increase the sampling frequency while obtaining the accurate pressure distribution and respiratory waveform from the smart mat system. For achieving this goal, the compressed sensing (CS) method was applied to our system. In [22], Donoho proposed a theory called compressed sensing which was considered as the first appearance of the theoretical framework of CS. Then Candes and Tao made a large contribution for perfecting the theory, and proved that the near-optimal signal can be recovered from random projections [23,24], and the process of signal recovery can be dealt with solving linear programming problems. Baraniuk further explained the CS theory and proposed a prototype called “single-pixel” compressive digital camera which applied the CS theory [25,26]. After more than ten years of development of CS, it can be demonstrated that signals can be compressed during the process of sampling with a frequency far below the Nyquist frequency, in other words, CS theory made the sampling and compressing in one procedure. So the data transfer efficiency will be greatly increasing through a low sampling time for one epoch data by using CS theory which ensures the accuracy of the data, even if the amount of data is very large. And in the recent years, the CS theory has promising application prospects in high resolution image acquisition such as MR image reconstruction [27,28,29], cryptology [30,31] and astronomical observations [32,33] etc. In addition to these application, the CS theory also have the potential ability to recognize the verbal labels contained in the large quantity of tactile patterns in the field of tactile sensing in human-robot interaction [21,34] .

The classic image compression technique is very convenient and practical for processing the common images, however, as for our application, CS method has an advantage of compressing the image during collecting the data, which means the compressed process is easily realized in the hardware which can reduce the sampling time. As for the classic image processing hardware such as the OV7620 or other integrated chips, they are used for compressing the classic images instead of pressure distribution. Thus the current commercial hardware are difficult to combine with our system implementation and not helpful for reducing the sampling time.

This paper mainly described a novel method for reducing the sampling time of proposed smart mat system with pressure sensor array by combining the CS theory, and the experiments for verifying the performance of the system with CS based method were conducted, and they were organized as follows. In Section 2, the system design, system implementation process and the way of generating the pressure distribution image and respiratory waveform from the system were proposed. In Section 3, the method focused on reducing the sampling time was presented, for the hardware part, the random measurement matrix was applied in sub-sampling process for acquiring few pressure points, and for the software part, the CS theory was utilized for reconstructing the original signals such as the pressure distribution image and respiratory waveform. To validate the performance of the improved system, the experiments were conducted. Section 4 presents experimental results to calculate the error rate of the reconstructed pressure distribution images, the correlation between the original respiratory waveform and reconstructed respiratory waveform and the accuracy of the respiratory rate. And in Section 5, the discussion with regard to the experimental results and the system performance was presented. In the last section, we summarize this paper.

## 2. System Implementation

This section proposed a technical solution and concept of a smart mat embedded with pressure sensor array to receive the body pressure distribution images and respiratory waveform for extracting useful features for medical analysis.

### 2.1. System Architecture

**Figure 1:**
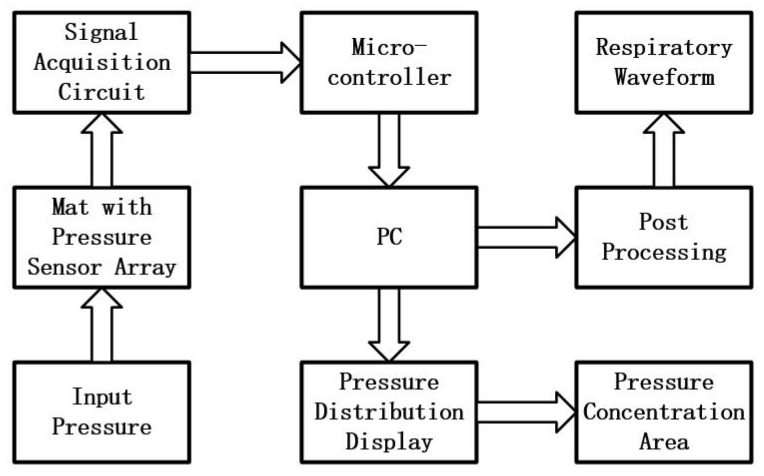
The framework of the system.

The goal is to develop a smart mat system to unobtrusively acquire the physiological information like respiration and pressure distribution of the subject. The whole system mainly includes a smart mat embedded with a pressure sensor array, the hardware circuit for acquiring the pressure values and the software for signal processing. The overall system framework is presented in Figure 1. The developed pressure sensor array is able to measure raw pressure values of a subject while lay on it. The signal acquisition circuit can gather pressure values from all pressure sensors, then the pressure signals acquired can be transmitted to the PC. In addition, many features which reflected the physiological information such as the respiratory waveform and pressure concentration area can be extracted from the pressure signals for medical analysis after post-processing.

### 2.2. Pressure Acquisition Circuit Design

**Figure 2:**
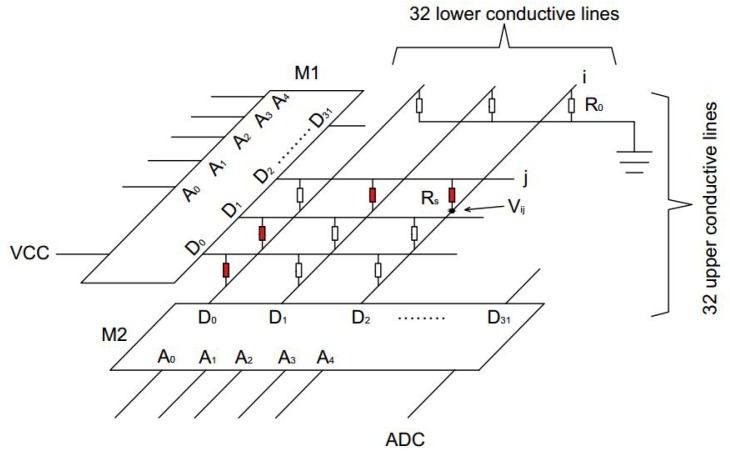
The schematic of pressure acquisition circuit.

In order to obtain the high-resolution pressure distribution and other information contained in it, a large quantity of pressure sensors are implemented. In addition, for minimizing the I/O pins of the hardware circuit, a sandwiched structure consisting of three layers were utilized [16]: A 32×32 pressure sensor array layer and two conductive layers. The schematic of the acquisition circuit is presented in Figure 2.

As for the pressure acquisition circuit, each conductive line on the upper conductive layer is connected to a voltage supply $V_{cc}$ via an analog multiplexer M1, and the conductive lines on the lower conductive layer are connected to an ADC via an analog multiplexer M2, and an offset resistor $R_{0}$ to the ground. There are 32 parallel conductive lines on the upper and lower conductive layers, respectively, while the conductive lines on the lower layer are vertical to the upper’s.

With the structure mentioned above, there is a sensor which is equivalent to a resistor at each intersection of two conductive lines of the upper and lower conductive layer, 1024 in total, so the voltage of each sensor can be acquired which indicates the pressure applied on it. In addition, the two multiplexers M1 and M2 are synchronously controlled by the processor in order to acquire the pressure applied on any sensor. For example, when M1 selected the conductive line *i* on the upper layer to $V_{cc}$ and M2 selected the conductive line *j* on the lower layer to the ADC, the voltage $V_{ij}$ in the row *i* and column *j* can be acquired through the ADC. By applying the scanning circuit, we can acquire the pressure signal of any part of the mat randomly by controlling M1 and M2. For example, the sensors with red mark can be selected to connect the circuit and feedback the pressure values, and the others would not feedback as shown in Figure 2.

### 2.3. Smart Mat Structure

**Figure 3:**
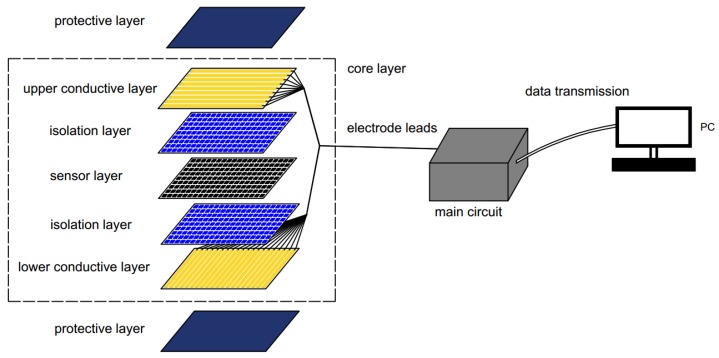
System overview.

Figure 3 showed an overview of the novel proposed smart mat system. In the system, a seven-layer structure was adopted. The upper and lower conductive layers conducted each sensor and transferred the signals to the signal acquisition circuit. The sensor layer was embedded with a pressure sensor array. The upper layer, lower layer and sensor layer are the most important part in the structure, and the three layers can be called “core layer”. The protective layers were used for protecting the “core layer” and preventing the electrical device from contacting directly with the external environment. The style of the protective layer can be the bed mat, swaddling and the cushion and the “core layer” can be sandwiched into those objects to become “smart swaddling” or “smart cushion”. The isolation layers were utilized to avoid the influence of gravity caused by the structure itself. And the isolation layers could be optional parts, and if the gap between the conductive layers and the sensor layer is too small, the isolation layers were required.

### 2.4. Hardware Implementation and Prototype

**Figure 4:**
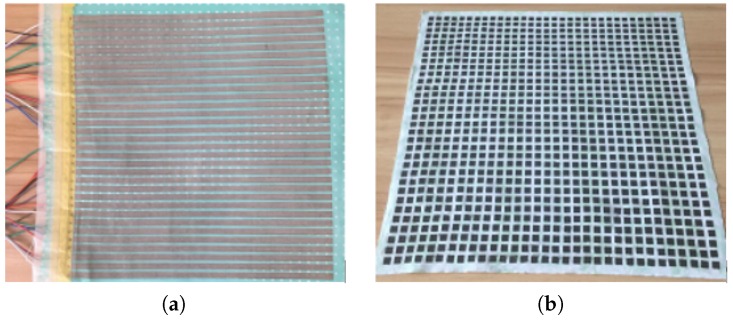
(**a**) The conductive layer. (**b**) Sensor layer.

As previously mentioned, the goal of this specialized smart mat is to record the pressure distribution and pressure changes of body while the subject laying on the mat without any interference, so the whole smart mat is made of soft textile materials. And the materials of the mat can be adapted to suit for various monitoring environments. The “core layer” is less than 2 mm in thickness, and the upper conductive layer is made of cotton cloth on which is printed with 32 parallel conductive lines which made of silver powder by applying the screen printing technology, the interval between two adjacent conductive lines is 3 mm and the width of each conductive line is 6 mm. The lower conductive layer is the same with the upper conductive layer, but its conductive lines are put vertically to the upper 32 lines. Figure 4a showed the picture of the conductive layer. The sensor layer is a linen cloth embedded with a 32×32 pressure sensor array as shown in Figure 4b. The pressure sensor is made of a kind of flexible material called “velostat” which is less than 0.1mm in thickness [35] . Each pressure sensor is a 6 mm × 6 mm square, and the interval of two sensors is 3 mm, same with the conductive line interval. The initial resistance of each pressure sensor is really high when no pressure applied on it. However, the resistance of the pressure sensor became smaller in millisecond with the force applied and restored immediately with the force removed. So the response time and relaxation time are at the level of millisecond. And the resistance values can range from about 50 Ω to 250 KΩ for a single small sensor with the dimension of 6 mm × 6 mm. The average human body pressure applied on the mat ranged from 0.15 × 10${}^{4}$ N to 0.4 × 10${}^{4}$ N, and the pressure sensor is very sensitive for this range. In that case, the sensor can sense the pressure change caused by body applied on it. Sensor array is composed by the single sensors, thus has the same performance as a single sensor. The pressure sensors and the square-holes in sensing layer are cut by program controlled laser cutting machine, so the sensor array is stable and re-producible.

**Figure 5:**
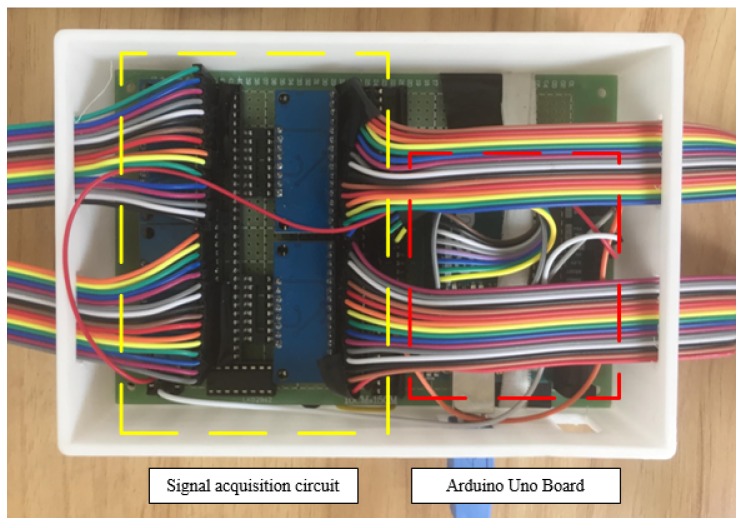
Signal acquisition circuit and Arduino Uno Board.

The microcontroller used in the system is an Arduino Uno [36], and the signal acquisition circuit and Arduino Uno board are presented in Figure 5.

**Figure 6:**
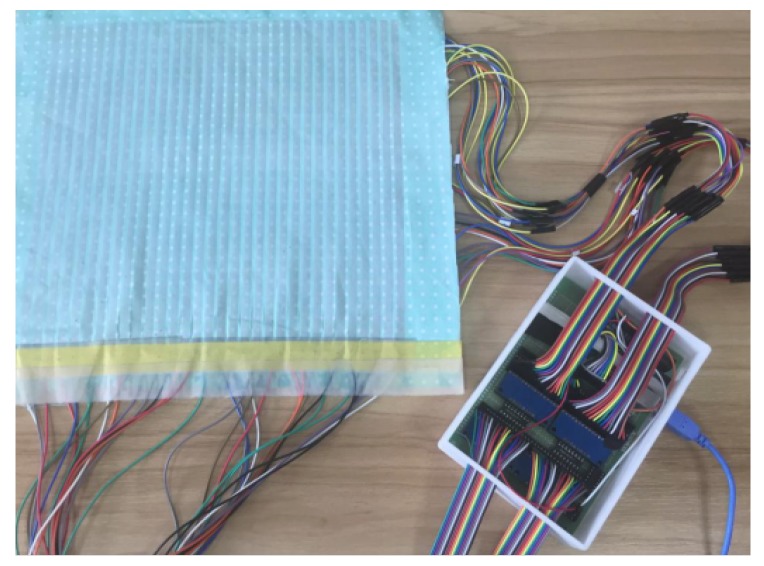
Prototype.

The prototype is shown in Figure 6, the area generating the pressure image with the size of 38.5 cm long and 36 cm wide is approximately the same size as an adult’s chest.

### 2.5. The Data Processing of the System

**Figure 7:**
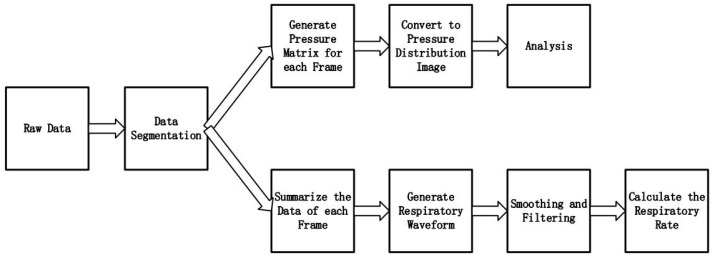
Data processing flow.

**Figure 8:**
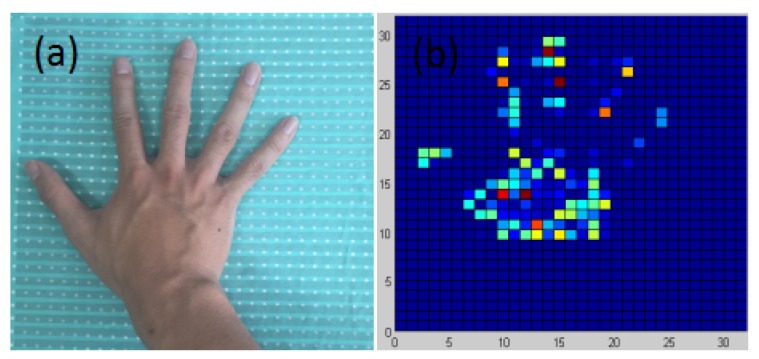
(**a**) A hand pressed on the prototype (**b**) The pressure distribution image of the hand.

The data processing flow chart was shown in Figure 7, when prototype was under pressure, all 1024 sensors can generate 1024 voltage values continuously which composed one frame of pressure image. So the raw data should be segmented into a large number of frames, and each frame data containing 1024 pressure values can be converted into a 32×32 pressure matrix. Those voltage values in pressure matrix between 0 from +5 V were normalized to 0–255, and the blue and red in color map represented 0 and 255 respectively, then the pressure distribution images for following analysis were transformed from the pressure matrix. Due to the high resolution of pressure sensor array, this prototype can clearly visualize the details of the pressure distribution and benefit the post analysis as described in our previous study [19]. For example, when the hand was put on the prototype, the computer can do the process and display the pressure distribution image as shown in Figure 8.

Except for generating the pressure distribution image, we also applied this system to measure the respiratory waveform of subject by detecting the pressure changes due to respiration as shown in Figure 7. When subject lay on the bed, the prototype was put under the trunk part of subject to record the pressure changes, and the alternating respiratory movements were large enough to cause a substantial change in the total pressure of the mat area. So we can plot the respiratory waveform by adding all 1024 pressure values in one frame as one point in the waveform.

## 3. Method

In this section, a novel method based on the theory known as compressed sensing (compressive sampling) [22,25,37] which can reconstruct the original signals from few sampling points instead of all 1024 sampling points was exploited .

### 3.1. The Sparsity of the Body Part Pressure Distribution Images in 2D-DCT Transform Domain

This method based on CS is suitable for the recovery of sparse signals, however, many useful signals in this world are not sparse itself, but fortunately those signals can always be sparse in other transform domains. The more sparse the signal is, it means the more concentrated information it contains. In [38], Candes et al. proposed that if {ξ${}_{1}$, ... , ξ${}_{m}$} were chosen from {x${}_{1}$, x${}_{2}$, ..., x${}_{n}$} randomly, where m ≪ n, then with high probability, every sparse signal {f(x) ; x = 1, 2 , ... , n} with *S* nonzero items can be recovered from f(ξ${}_{1}$), . . . , f (ξ${}_{m}$), so long as m ≥ C*Slog* n for some absolute constant C, and numerical experiments suggest that most S-sparse signals can be recovered exactly once m ≥ 4*S* or so. So the test of calculating the sparsity of the signal must be conducted to verify the feasibility of the method.

In our system, the original signal is a 32 × 32 pressure distribution image contained 1024 pixels, and as a two-dimensional signal, many transforms can be utilized for converting the original non-sparse signal to sparse signal such as two-dimension discrete Fourier transform (2D-DFT), two-dimension discrete cosine transform (2D-DCT), two-dimension wavelet transform and so on. The 2D-DCT is commonly used in graphic compression algorithms [39], which can concentrate most information in a few frequency coefficients. In this paper, 2D-DCT was employed to obtain the sparse expression of original signal due to its advantages for processing with this condition. The 2D-DCT transform can be represented as the following form [40]:

(1) $$F\left(u,v\right)=\alpha \left(u\right)\beta \left(v\right)\sum_{x=1}^{P}\sum_{y=1}^{Q}\cos\left[\frac{\pi (2x-1)(u-1)}{2P}\right]\cos\left[\frac{\pi (2y-1)(v-1)}{2Q}\right];$$

where x∈{1,2,...,P} and y∈{1,2,...,Q} are the coordinates sets of two-dimensional function f(x,y), and {F(u,v); u = 1,2,...,P, v = 1,2,...,Q} is the set of 2D-DCT coefficients, and:

(2) $$\alpha \left(u\right)=\left\{\begin{array}{cc} \sqrt{1/P} & \left(u=1\right)\\ \sqrt{2/P} & \left(2\leq u\leq P\right) \end{array}\right.$$

(3) $$\beta \left(v\right)=\left\{\begin{array}{cc} \sqrt{1/Q} & \left(v=1\right)\\ \sqrt{2/Q} & \left(2\leq v\leq P\right) \end{array}\right.$$

And if the sampling values {*f*(x, y); x = 1, 2,...,P, y = 1,2,...,Q} and their corresponding coordinate positions are obtained, the 2D-DCT coefficients {*F(u, v)*; *u* = 1,2,...,P, *v* = 1,2,...,Q} are uniquely determined, on the contrary, if the 2D-DCT coefficients are known, the sampling values could be uniquely determined, too. To acquire the sparse degree of the pressure distribution images produced by our smart mat system, 2613 pressure distribution images of the body parts were converted to 2D-DCT domain, and the average maximum 2D-DCT coefficient of the 2613 images was 2.85 × 10${}^{3}$. Although the sparsity means the number of non-zero coefficients, practically, the coefficients could be considered to be zero if they were small enough. So the small coefficients which were less than 5%, 4%, 3%, 2%, 1% of average maximum coefficient were considered as zero, respectively, and the sparsity of signals after 2D-DCT can be computed correspondingly. The results were presented in Table 1.

From Table 1, it is demonstrated that the least sampling points is very small due to the sparsity of the transformed signal, thus saved a large amount of sampling time. Obviously, the more sampling points we adopt, the more accurate the recovered signals are, thus we need to balance the sampling time and recovery accuracy, so the number of sampling points will also be studied in our following research.

### 3.2. The Principle of Compressed Sensing

The CS theory can accurately recover sparse signal from limited random measurements, and the sparsity of the pressure distribution images tested before had been proved to satisfy the requirement of reconstruction of pressure distribution image by applying the CS theory. The process of recovering or reconstructing the sparse signals can be described in mathematical expression as follows, the signal to be reconstructed is {f*(x, y); x = 1,2,...,P, y = 1,2,...,Q; N = PQ} and the few random measurements is {*f*(x${}_{m}$, y${}_{m}$); m = 1,2,...,M}, where {(x${}_{m}$, y${}_{m}$); m = 1,2,...,M} means the location of sample values *f*(x${}_{m}$, y${}_{m}$), and the M ≪ N. The objective is to accurately reconstruct f*(x, y) from *f*(x${}_{m}$, y${}_{m}$), for achieving this goal, we can use the following linear equation:

(4) Az=b

(5) $$A=\left[\begin{array}{ccccccccc} A_{1,1,1} & A_{1,1,2} & \cdots \; & A_{1,1,Q} & A_{1,2,1} & \cdots \; & A_{1,2,Q} & \cdots \; & A_{1,P,Q}\\ A_{2,1,1} & A_{2,1,2} & \cdots \; & A_{2,1,Q} & A_{2,2,1} & \cdots \; & A_{2,2,Q} & \cdots \; & A_{2,P,Q}\\ \vdots & \vdots & \ddots & \vdots & \vdots & \ddots & \vdots & \ddots & \vdots \\ A_{M,1,1} & A_{M,1,2} & \cdots \; & A_{M,1,Q} & A_{M,2,1} & \cdots \; & A_{M,2,Q} & \cdots \; & A_{M,P,Q} \end{array}\right]$$

where

(6) $$A_{m,u,v}=\alpha \left(u\right)\beta \left(v\right)\cos\left[\frac{\pi \left(2x_{m}-1\right)\left(u-1\right)}{2P}\right]\cos\left[\frac{\pi \left(2y_{m}-1\right)\left(v-1\right)}{2Q}\right]$$

(7) $$z^{T}=\left[\begin{array}{ccccccccc} F(1,1) & F(1,2) & \cdots \; & F(1,Q) & F(2,1) & \cdots \; & F(2,Q) & \cdots \; & F(P,Q) \end{array}\right]$$

(8) $$b=\left[\begin{array}{cccc} f(x_{1},x_{1}) & f(x_{2},x_{2}) & \cdots \; & f(x_{M},x_{M}) \end{array}\right]$$

Matrix ***A*** is called sensing matrix ( M×N ) which is constructed by multiplying the measurement matrix Φ and transform basis Ψ , as the following form:

(9) A=ΦΨ

The measurement matrix or called random observation matrix Φ can be realized by the hardware of acquisition system and to provide the location information of random measurement points. The form of transform basis Ψ depends on the selected transform domain. Hence the component $A_{m,u,v}$ in the ***A*** contained the information of the location and the transform domain of *m*th random measurement as shown in the equation (6). Vector ***z*** presented the sparse signal (N × 1 column vector) to be recovered and it is 2D-DCT coefficients sequence {*F (u, v)*; *u* = 1, 2,...,P, *v* = 1,2,...,Q} in this paper. Vector ***b*** presented the sequence of random limited measurements (M × 1 column vector). If the transform domain and sampling points (random measurements) were determined, the ***z*** could be obtained by solving the linear equation (4). Once the ***z*** are known, the object f*(x, y) could be calculated by 2D-IDCT from ***z***.

The linear equation ***Az*** = ***b*** cannot be solved directly because the row number of matrix ***A*** is less than the the length of vector ***b*** (M < N), in other words, the equation is underdetermined. Although the equation ***Az*** = ***b*** cannot be solved directly, the prior information contained in the limited random measurements such as the sparse distribution of 2D-DCT transform domain will help us reconstruct the original signal with great probability. Candes proved that if the sensing matrix ***A*** satisfies the restricted isometry property (RIP), the solution of the underdetermined equation ***Az*** = ***b*** is equivalent to find out the optimal solution in following conditions [23]:

(10) $${min∥z∥}_{0}\;s.t.\;\mathrm{Az}=b$$

But the process of finding the minimum of ${∥z∥}_{0}$ is difficult to accomplish in polynomial time, namely, the problem of seeking minimum of ${∥z∥}_{0}$ is a NP-hard problem. The essence of finding the least ${∥z∥}_{0}$ is to discover the non-zero coefficient in ***z***, and Candes claimed that if the sensing matrix ***A*** satisfied a more stringent RIP condition [23], the solution of (10) is equivalent to the solution of (11).

(11) $${min∥z∥}_{1}\;s.t.\;\mathrm{Az}=b$$

The problem of solving the least ${∥z∥}_{1}$ could convert to a convex optimization problem and the optimal solution could be found out in a polynomial time.

### 3.3. Solving the Least 1 Norm Problem

#### 3.3.1. Transformation of the Least 1 Norm

As we know, the ${∥z∥}_{1}$ can be expressed as $\sum_{i=1}^{}\left|z_{i}\right|$, but this problem is non-differentiable in its feasible region, so this problem cannot be directly solved by classical convex optimization method. Therefore a conversion process should be further introduced to make this problem become a convex optimization problem. The way to make ${∥z∥}_{1}$ differentiable is to introduce a set of slack variables {$\mu_{i}$; i = 1,2,...,N} and for every $z_{i}$ and $\mu_{i}$ have the relation as the following form [41]:

(12) $$-\mu_{i}\leq z_{i}\leq \mu_{i}$$

Then the original problem can keep away from the non-differentiable point in the feasible region with the help of slack variables. Rewrite (11) as the following form:

(13) $$\begin{array}{c} min\sum_{i=1}^{}\mu_{i}\\ s.t.\;z_{i}-\mu_{i}\leq 0\\ \;-z_{i}-\mu_{i}\leq 0\;\left(i=1,2,\ldots ,N\right)\\ \;\mathrm{Az}=b \end{array}$$

Suppose $f_{0}\left(z\right)$ denotes the objective function $\sum_{i=1}\mu_{i}$ , {$f_{l}=\left(z\right)$ ; *l* = 1,2} denotes two inequality constraints, and { $h_{k}=\left(z\right)$; k=1 }denotes the only equality constraint in this problem, so $f_{0}\left(z\right)$, $f_{l}\left(z\right)$, $h_{k}\left(z\right)$ can be written as the following form:

(14) $$\begin{array}{c} f_{0}\left(z\right)=1^{T}\mu \\ f_{1}\left(z\right)=z_{i}-\mu_{i}\\ f_{2}\left(z\right)=-z_{i}-\mu_{i}\\ h_{1}\left(z\right)=\mathrm{Az}-b \end{array}$$

#### 3.3.2. Prime-Dual Interior Method

The new constrained problem (13) is a classical convex optimization problem and could be solved by prime-dual interior point method [42]. The prime-dual interior point method was a developed convex optimization algorithm, and it has been applied for recovering the original signal based on CS theory for more than ten years. The advantage of this method is that the accuracy of the recovered signal can achieve a high level and it can also handle with big data such as MRI images. The prime-dual interior point method is to find the optimal solution by constantly narrowing the dual gap between the feasible solution of prime problem and dual problem which derived from the prime problem. Suppose the dual problem can be denoted as the following form:

(15) maxθ(λ,ν)s.t.λ>0

Combined with the prime problem and dual problem, the Karush-Kuhn-Tucker conditions (KKT) of the two problems can be used to solve the optimization problem, they are the necessary and sufficient conditions for optimality and imply that the dual optimum is attained if the optimal duality gap is zero. So if (z,λ,ν) satisfies the KKT conditions, z is also optimal [42]. The KKT conditions in this problem were demonstrated in the following relations:

(16) $$\begin{array}{c} f_{l}\left(z^{*}\right)\leq 0,l=1,2\\ \mathrm{Az}-b=0\\ \lambda_{l}^{*}\geq 0\\ \lambda_{l}^{*}f_{l}\left(z^{*}\right)=0,\\ ▽f_{0}\left(z^{*}\right)+\sum_{l=1}^{2}\lambda_{l}^{*}▽f_{l}\left(z^{*}\right)+A^{T}v^{*} \end{array}$$

where $z^{*}$ presented the optimal solution of prime problem, and $\left(\lambda^{*},\nu^{*}\right)$ present the optimal solution of dual problem. The KKT conditions play an important role in optimization, many algorithms for convex optimization are conceived as, or can be interpreted as, methods for solving the KKT conditions [42].

#### 3.3.3. Newton Method for Searching the Optimal Solution

The procedure of finding optimal solution can apply the classical Newton method. Suppose a point $a_{0}=\left(z^{0},\lambda^{0},\nu^{0}\right)$ of the prime-dual problem as the initial point, the search direction as △a=(△z,△λ,△ν). In practice, we relax the complementary slackness condition $\lambda_{l}^{*}f_{l}\left(z^{*}\right)=0$ to $\lambda_{l}^{*}f_{l}\left(z^{*}\right)=-1/\tau$, and the parameter τ will increase dramatically as the number of iterations increases, so when the iterative process terminates, the equation $\lambda_{l}^{*}f_{l}\left(z^{*}\right)=-1/\tau$ is very similar to $\lambda_{l}^{*}f_{l}\left(z^{*}\right)=0$. The advantage of relaxing the complementary slackness condition is that the search path will be smooth and facilitating the process of the algorithm [43,44]. Define the residual matrix $r_{\tau }\left(z,\lambda ,\nu \right)$ as the following form:

(17) $$r_{\tau }\left(z,\lambda ,\nu \right)=\left[\begin{array}{c} ▽f_{0}\left(z\right)+Df{\left(z\right)}^{T}\lambda +A^{T}v\\ -diag(\lambda )f(z)-(1/\tau )\\ \mathrm{Az}-b \end{array}\right]$$

where the three parts of this matrix were three equality constraints in the KKT conditions, respectively.

If the point $a^{*}=\left(z^{*},\lambda^{*},\nu^{*}\right)$ is the optimal solution of the problem, the residual matrix $r_{\tau }\left(z^{*},\lambda^{*},\nu^{*}\right)=0$. And if $r_{\tau }\left(a^{0}+△a\right)=0$, △a=(△z,△λ,△ν) will be the direction of the location of optimum solution from the initial point $a^{0}$. Then move forward in the direction △a=(△z,△λ,△ν) with a search step *s*, the point $\left(a^{0}+s△a\right)$ will get closer to the optimum solution. But this method cannot find the optimum solution at once because the complementary slackness condition was further relaxed in this problem. Hence in each iteration, the new search direction were calculated based on the sub-optimum point of last iteration and the step *s* was updated for finding the new start point of next iteration. And the solution in this iteration could be considered the optimal solution until the duality gap between the prime and dual problem was less than a specified value or the iterations reach a certain number of times.

### 3.4. The Realization of Measurement Matrix in Hardware

In order to reconstruct the pressure distribution image and respiratory waveform, the measurement matrix is actually realized in the hardware part which sent M voltages and their corresponding locations to the PC. Actually, those locations were expression form which denoted the measurement matrix Φ. The random measurements {($x_{m},y_{m}$) ; m = 1, 2, ..., M } are corresponding to the M × N-dimensional matrix Φ, and Φ was merged with the transform basis Ψ to become the sensing matrix A as shown in Equations (5) and (9). The RIP condition which should be satisfied by sensing matrix A has an important equivalent condition is that the Φ should be incoherent with Ψ, in other words, the rows in Φ and the columns in Ψ cannot be represented with each other [25]. This equivalent condition moves the “responsibility” of meeting the RIP condition from sensing matrix A to the measurement matrix Φ due to the invariance and orthogonality of the transform basis Ψ, so the performance of Φ influences the realizability and consequence of the signal recovery.

In our system, the random observation matrix is produced with the help of the pseudorandom number generator in arduino 1.6.12, and the pseudorandom number satisfies the Gaussian distribution. So the measurement matrix Φ is Gaussian random measurement, and the process of generating one random number is to generate a N-dimension row vector of Φ, then the random number was converted to binary numbers which was used to control the multiplexers as an address signal. It should be noted that in order to construct the measurement matrix Φ, the address should be transmitted to the PC alongside with the pressure value.

### 3.5. The Novel Framework of the System with CS Method

**Figure 9:**
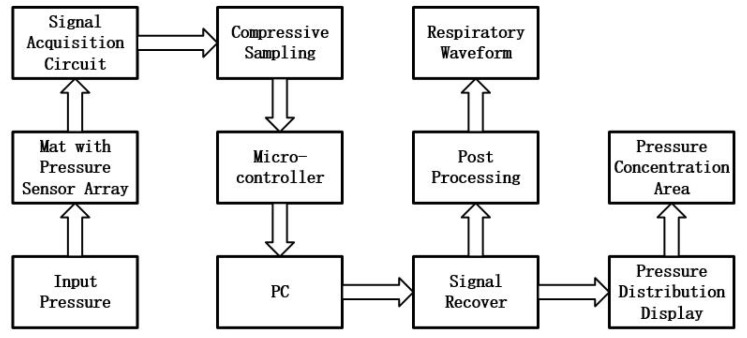
The novel framework of the system.

The novel framework of the system with sub-sampling method and reconstruction algorithm was shown in Figure 9. The main difference between the original framework shown in Figure 1 and the novel framework shown in Figure 9 was that the latter has two added processes: compressive sampling and signal recovery. The signal acquisition circuit gather partial pressure points randomly instead of all points. Then the original pressure signals can be reconstructed via the recover algorithm, then the reconstructed pressure signals would be processed as same as the original as shown in Figure 1 .

**Figure 10:**
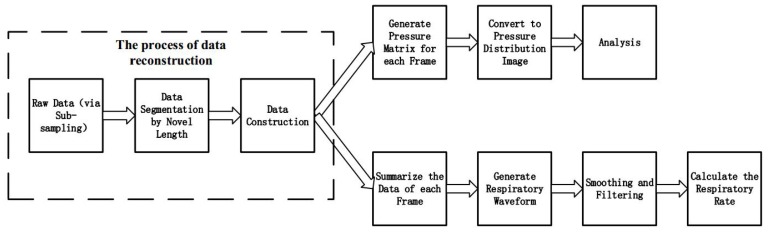
Data processing flow with the process of data reconstruction.

**Figure 11:**
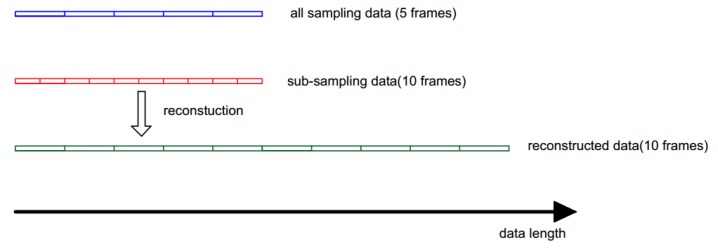
The data length before and after the reconstruction for sub-sampling data.

The novel data processing flowchart was shown in Figure 10. For previous system, 1024 pressure values consist one frame data, but the novel method made very few number of pressure values as one frame. So the process of data segmentation should suit the frame with novel length, then the pressure values whose number was less than 1024 in each frame were used to recover the original signal. Compared with the all sampling method, the novel method can transmit more frames with the same length data, and such a process can be expressed by Figure 11. The data flow drawn by blue line has 5 frames and the data flow drawn by red line has 10 frames, but the two data flows have the same length because the second data flow was acquired by the sub-sampling method. After the reconstruction, each frame in sub-sampling data flow become the one have original data length. The significance of this process lies in the fact that the same length of the data flow can produce more frames with high accuracy, in other words, the same information can be expressed by less data than before, so it raised the sampling frequency of the system.

## 4. Experiments and Results

In this section, the experiments of reconstructing the pressure distribution and respiratory waveform were conducted to verify the performance of the CS method applied to our system while the interpolation method was also applied for comparison.

### 4.1. Experiment Setup

**Figure 12:**
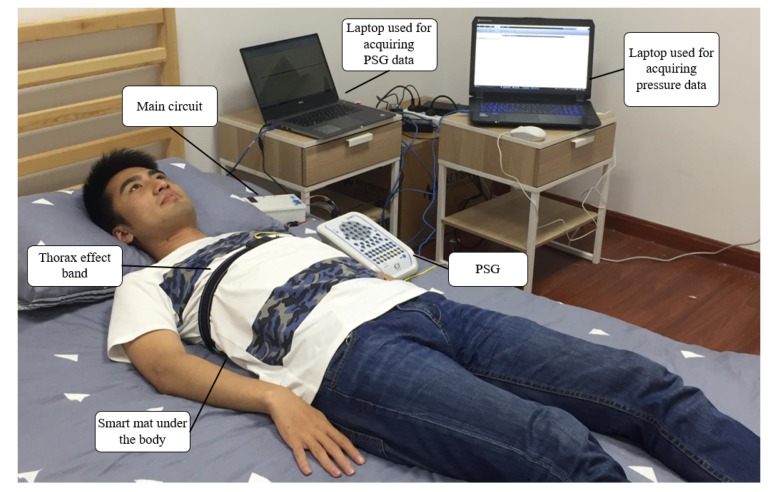
Experiment setup.

As a prototype of the smart mat, it was placed under the human body when the subject lay on the bed as shown in Figure 12. To obtain the respiration condition, Compumedics Grael PSG system was also applied to simultaneously record the thorax effort signal as a reference.

**Figure 13:**
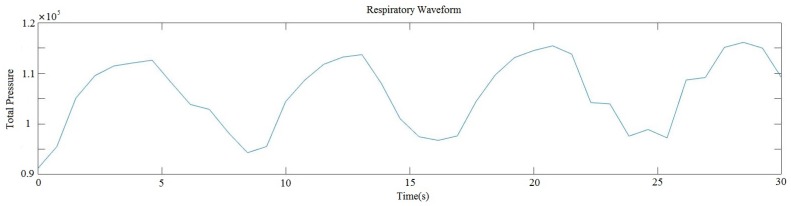
The respiratory waveform generated by the prototype.

For example, the respiratory waveform generated by this way was shown in Figure 13, the abscissa axis and the vertical axis in the coordinate system presented the time and the total pressure of all 1024 sensors, respectively. And the change of the total pressure was caused by the alternating respiratory movements and the noise. Due to the high sensitivity of pressure sensors, the voltage of single pressure point would vary caused by the noise and slight body movement.

The experimental results demonstrated that this system would consume about 0.2 s to gather all 1024 pressure values, in other words, it need about 0.2 s to generate one frame. The time consumption was much faster than our previous work mentioned in [19] due to the modified signal acquisition program, but the program was not mentioned in this paper because it’s not the emphasis of this study.

### 4.2. The Ability for the Reconstructing the Pressure Distribution Image

**Figure 14:**
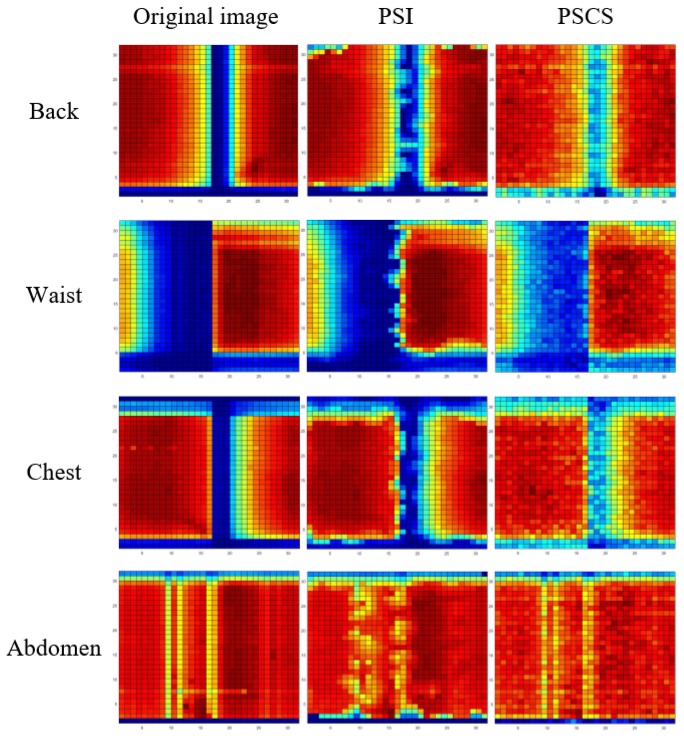
The pressure distribution images of different body parts generated by original, PSI and PSCS method, the latter two method used only 300 pressure points.

The pressure distribution image was obtained when the object placed on the mat, and the body part where used to extract respiratory waveform is also the object for the smart mat. The back, chest, abdomen and waist area are the main parts to extract the respiratory waveform in this system. And those areas are suitable for taking advantage of the CS method because those areas have low sparsity in transform domain. For obtaining the performance of the CS method, the pressure distribution images reconstructed by the CS method were compared with the images reconstructed by the interpolation method [45] and the groundtruth. The interpolation method utilized linear interpolation based on triangles, and the groundtruth was the image generated by all sampling points. As for example, we compared the results produced by the interpolation and CS method which utilized only 300 points randomly selected from the original 1024 points by Latin hypercube sampling algorithm operated by Matlab 2016a [46]. And the CS and the interpolation method used with this post-sampling points can be named post-sampling CS method (PSCS) and post-sampling interpolation method (PSI). Figure 14 showed the comparative results of the pressure distribution images of different body parts by the three different methods mentioned above, namely the original, PSI and PSCS method results.

As shown in Figure 14, the four rows denote the pressure distribution images of different areas namely back, waist, chest and abdomen while the columns denote the results from different methods namely the original, PSI method and PSCS method. The profile of the images reconstructed by the PSCS method were more similar to the original images, and the area where concentrates the large pressure is relatively precise. The images reconstructed by PSI method were warped in the edge between high pressure area and low pressure area, resulting that these images cannot describe the actual body profile. But the PSCS method was weak in dealing with the area composed by amounts of zero due to the zero data would be replaced by very small data leading to the phenomenon that the light blue blocks emerged in the area without pressure. Fortunately, the algorithm can be modified to eliminate this phenomenon.

The accuracy of the image reconstructed influenced the qualities of the physiological features extracted from the body pressure distribution, so a quantitative assessment which can estimate whether the reconstructed images are accurate enough to reflect the ground truth of the pressure distribution and extract useful physiological features, is required. For example, the error rate of the images reconstructed by two methods can quantitatively estimate the accuracy of the image reconstructed. Define the error rate for estimating the recover performance of those images as the following form:

(18) $$Error\;rate\;of\;images=\frac{1}{N}\sum_{i=1}^{N}\sqrt{\frac{\sum_{x=1}^{P}\sum_{y=1}^{Q}{\left[f^{*}\left(x,y\right)-f\left(x,y\right)\right]}^{2}}{\sum_{x=1}^{P}\sum_{y=1}^{Q}{\left[f\left(x,y\right)\right]}^{2}}}$$

where f(x,y) and $f^{*}\left(x,y\right)$ denote the true value of the groundtruth and the corresponding value estimated by reconstruction algorithms. In this system, P and Q value are 32 due to the structure of pressure sensor array. The N presents the total number of images for calculating the error rate of images. Table 2 showed the error rate calculated from 6142 images reconstructed by the PSI and the PSCS method when the number of the randomly sampled points ranged from 100 to 500.

**Figure 15:**
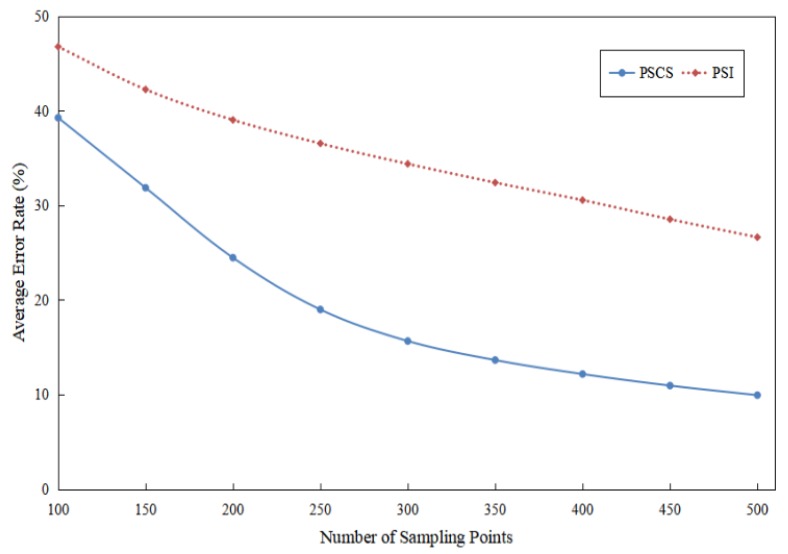
The change trend of average error rate for the PSI and the PSCS method with the number of randomly sampling points ranging from 100 to 500.

And Figure 15 showed the change trend of the average error rate for the PSI and the PSCS method when the number of randomly sampled points ranged from 100 to 500. From the change trend of two curves, the conclusion could be drawn in this experiment that the PSCS method performed better than the PSI method with regard to the error rate of reconstructed pressure distribution images.

### 4.3. The Ability for Reconstructing the Respiratory Waveform

**Figure 16:**
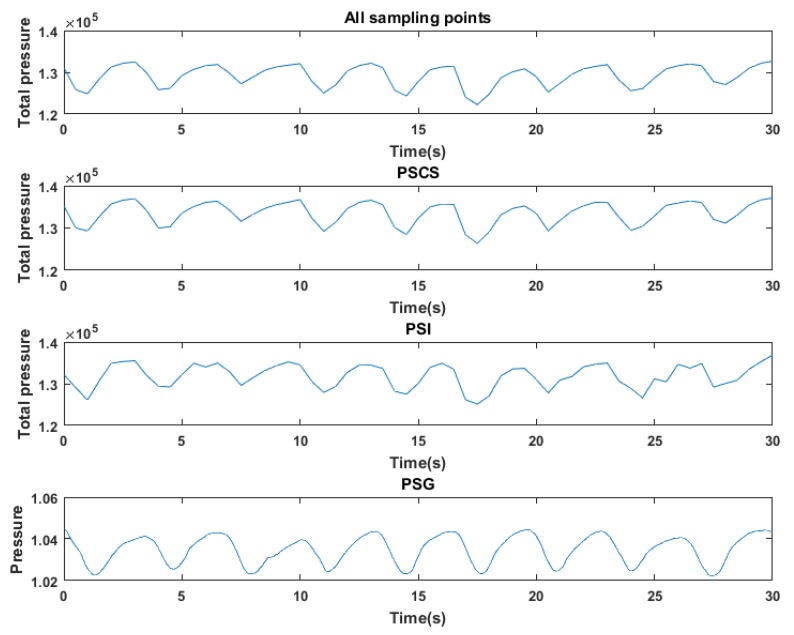
The respiratory waves produced by all sampling points, the PSCS method, the PSI method and the PSG system, the PSCS method and PSI method utilized 300 points randomly selected from the original 1024 points.

The experiment environment for obtaining the respiratory waveform was as same as the environment described in the Section 4.1. Taking the case that subject lay on the mat in a supine posture as the condition of experiment, the respiratory waveform generated by the all sampling points could be obtained. Meanwhile, PSG system was also applied to record the thorax effort signal simultaneously as a reference as shown in Figure 12. Then the randomly acquired pressure signals can be used for applying the PSI and the PSCS method to obtain the reconstructed respiratory waveform. The first row, second row, third row and fourth row in the Figure 16 demonstrated the respiratory waves produced by original method, the PSCS method, the PSI method and the PSG system. And the PSCS method and PSI method also utilized only 300 points randomly selected from the original 1024 points.

From the Figure 16, the difference between four respiratory waves can be clearly found. The processed PSG thorax effort signals in the fourth row demonstrated that the subject integrally breathes 9 times in 30-seceonds. The respiratory waves created by all sampling points also had about nine integrated valleys without any post-processes such as smooth or other filter algorithms. The respiratory wave in first row and the second row are quite similar, but the waveform in third row indicated that the PSI method performs poor in respiratory waveform recovery. Similar to the analysis processed in the previous section, the quantitative analysis of the reconstruction performance of the respiratory waveform is also needed. So the correlation between the reconstructed respiratory waves and the original waves which can reflects the capacity of the reconstruction method was calculated. Table 3 showed that 60 30-second epochs waves were used to calculate the average correlation between the waves reconstructed from the PSI method with the original waves, and the average correlation between the waves reconstructed from the PSCS method with the original waves. And the number of sampling points used for reconstruction ranged from 100 to 500.

**Figure 17:**
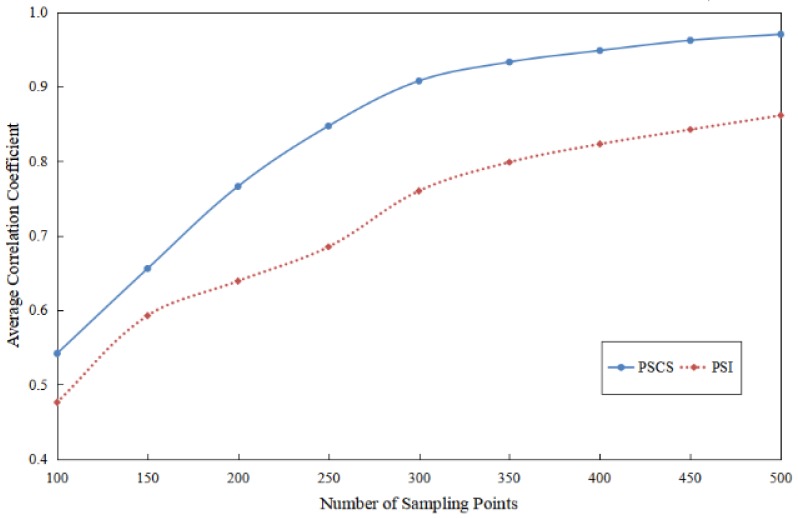
The change trend of the average correlation between the waves reconstructed from the PSI method with the original waves, and the average correlation between the waves reconstructed from the PSCS method with the original waves with different number of sampling points for 60 30-second epochs.

Figure 17 showed the change trend of the curves which described the two kinds of average correlation presented in Table 3. And from the change trend of two curves, we can draw the conclusion that the PSCS method had better performance than the PSI method in reconstructing the pattern of the respiratory waveforms in this experiment.

### 4.4. The Accuracy of the Respiratory Rate Extracted from the Respiratory Waveform Reconstructed by the Novel Method

Once the respiratory waveform generated, the information contained in the waveform could be obtained, and the respiratory rate (RR) is the one of the most important information. In general, the respiratory rate was calculated from the time interval between the adjacent end-inspiration in the respiratory waveform for a certain time which can be called one epoch. For this study, we set 30 s as one epoch and calculate its RR, because some medical analysis were generally conducted on 30 second epochs by medical experts or algorithms such as sleep analysis [47].

It is worth noting that with different conditions the subject lay on the mat, the peaks or valleys represented the end of inhalation correspondingly. As it is demonstrated in our previous study [19], when the subject lay on the mat with a supine posture and the back or waist part of body is in the measured area, the valleys in the respiratory waveform represented the end of inhalation. On the contrary, when the subject lay with a prone posture and the chest or abdomen part of body is in the measured area, the peaks represented the end of inhalation. The RR for each epoch could be given by

(19) $$INT_{i}=\frac{\sum_{j=1}^{n-1}\left(t_{i,j+1}-t_{i,j}\right)}{n-1}$$

(20) $$RR_{i}=\frac{60}{INT_{i}}$$

in which $t_{i,j}$ is the moment of *j*th valley of the ith epoch, and $INT_{i}$ is the mean valley interval for ith epoch, n is the number of valleys, so the respiratory rate could be demonstrated by $RR_{i}$. It’s worth noting that when we extract the RR from the respiratory waveform generated by the subject who lay with a supine posture, the waveform should be turned over to ensure that the valley present the end of inhalation. Actually, the PSG signals shown in this study were already processed by turning over.

The experiment results about the performance of the reconstruction of the pressure distribution images and the respiratory waveform in previous sections were obtained with the help of sampling all the pressure points (post-sampling method). However, our goal is to reduce the sampling time of the system by means of sampling partial pressure points randomly. So the process of directly sampling partial points of the sensors in the hardware as mentioned in Section 3.4 and then reconstructing the signals were necessary. And the points randomly sub-sampled in the hardware can be consider as fore-sampling, so the CS and the interpolation method used with those fore-sampling points can be named fore-sampling CS method (FSCS) and fore-sampling interpolation method (FSI). The main distinction between the PS and FS method is that the PS method actually sampled all 1024 points for each frame.

**Figure 18:**
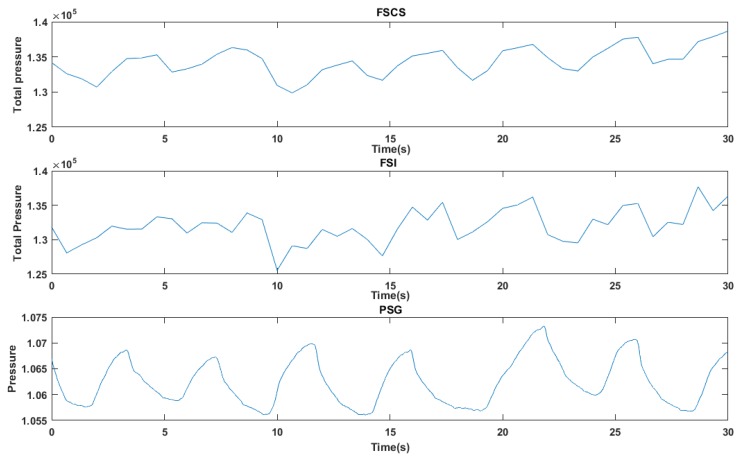
The respiratory waves produced by the FSCS method ,the FSI method and the PSG system.

In Figure 18, the first row and the second row showed the respiratory waveform reconstructed by the FSCS and the FSI method with 300 sampling points in one frame, respectively. And the third row showed the PSG thorax effort signal in the same 30-second epoch.

The respiratory waveform reconstructed by FSCS and FSI method in Figure 18 were more elaborate than the reconstructed waveform reconstructed by PSCS and PSI in Figure 16, such as the details like the small changes in the FSCS and FSI waves can be found, because the FS method can plot more points in the respiratory waveform with the same time duration. In addition, respiratory waveform produced though lower sampling time can contain higher frequency components. But the respiratory waveform needs further post-processing to extract more accurate change trend such as smoothing, filtering or other processing. And if the sampling rate is very low, the post-processing cannot obtain a good performance since the low sampling rate would already cause information loss.

**Figure 19:**
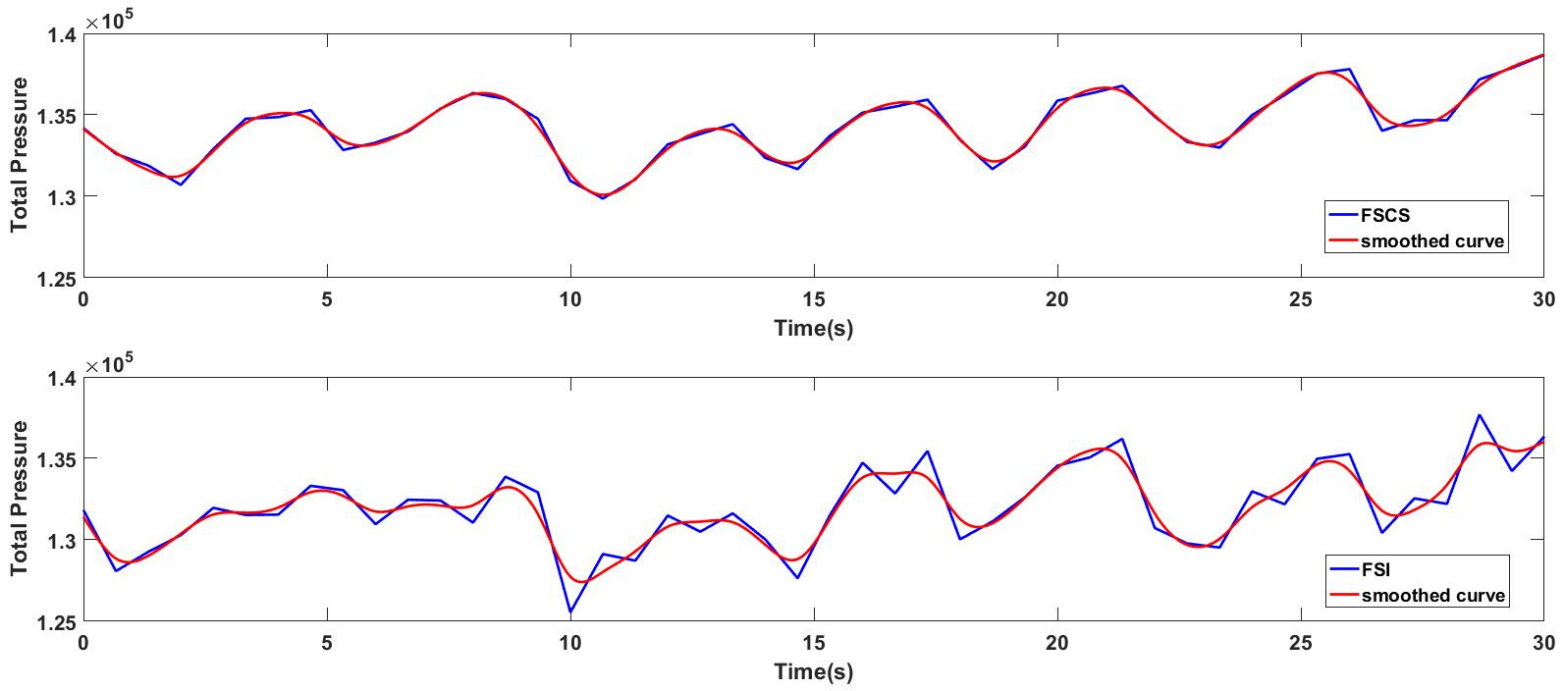
The respiratory waveform reconstructed by FSCS and FSI method before and after the smoothing process.

For obtaining the accuracy of the respiratory waveform which had relatively higher sampling frequency, the RR extracted from the respiratory waveform was utilized. Before the extraction of the RR, the respiratory waveform should be smoothed or filtered for valley or peak detection. For instance, Figure 19 showed the respiratory waveform of the same epoch pictured in Figure 18 reconstructed by FSCS and FSI method before and after the smoothing process. The blue solid line and the red solid line in Figure 19 represented the waveform before and after the smoothing for both methods, respectively. The smoothing process was realized by the curve fitting tools in Matlab2016a, and the smoothed curve was constituted of piecewise polynomial model which had the smoothing parameter p (p = 0.96812749). Obviously, the peaks in the smoothed curve reconstructed by FSCS method was more distinct than the FSI method. Especially for the first ten seconds in this epoch, the smoothed FSI curve was relatively flat, and this phenomenon made it difficult to extract the valleys and reflect the respiration trend of the subject. And the valleys in the smoothed curve reconstructed by the FSCS method can be easily detected.

Define the accuracy of RR for estimating the performance of extracting the RR from the reconstructed signals as the following form:

(21) $$Accuracy\;of\;RR=\frac{1}{N}\sum_{i=1}^{N}(1-\sqrt{\frac{{\left(RR_{i}^{C}-RR_{i}^{P}\right)}^{2}}{{\left(RR_{i}^{P}\right)}^{2}}})$$

in which $RR_{i}^{C}$ denotes the RR of ith epoch calculated by respiratory waveform reconstructed by the FS method, and the $RR_{i}^{P}$ denotes the RR of ith epoch calculated by PSG thorax effort signals. The *N* represents the total number of epochs used for calculating the accuracy of RR. Table 4 showed the accuracy of the RR of the respiratory waveform reconstructed by the FSI and FSCS method via five different sampling numbers, namely 100, 200, 300, 400 and 500, and each sampling number had 20 30-second epochs.

There are two main reasons why the accuracy of RR was considered as the performance measurement parameter, one is that the correlation between the reconstructed waves and groundtruth cannot be calculated because only few pressure points (100, 200, 300, 400, 500) were sampled instead of all 1024 pressure points which means the respiratory waveform produced by all the pressure sensors could not be acquired at the same time for comparison, and another reason is that the RR is an important physical sign containing much useful information.

### 4.5. The Time Consumption in Hardware after Using the FSCS Method

As mentioned above, one of the important reason for applying the sub-sampling method to reconstruct the pressure distribution image and respiratory waveform is to reduce the sampling time. But the time consumption for generating one frame is not linear to the number of sampling pressure points, because the locations of the sampling pressure points need to be sent to PC for reconstruction alongside with the pressure values. So the time of acquiring and sending both the pressure values and their location information for 512 points is close to the time of acquiring and sending all 1024 pressure values for using original method by using the hardware of this system. And starting from 512 points, each reduction of 100 sampling points will lead to the 20% reduction of the original sampling time. So when 300 points were randomly sampled, about 40% of the sampling time can be decreased on this system by utilizing the FSCS method.

As mentioned above, it will need nearly 0.2 s for sampling all pressure points in one frame, then based on the relationship between the number of reduced sampling points and the reduction of the original sampling time, it was easy to calculate the actual reduced time at different number of sampling points.

## 5. Discussion

As shown in Figure 17, the average correlation between the respiratory waveform reconstructed by the PSCS method and the original waveform was larger than the correlation between the waveform reconstructed by the PSI method and the original waveform. When 300 random points were used to reconstruct the respiratory waveform, the average correlation between the PSCS waveform and the original waveform was 0.9078, but 0.7601 for PSI waveform. The reason why the PSCS method can achieve better performance in reconstructing the signals is that the PSCS method has taken advantages of the information beyond those pressure values such as the information of transform domain and statistical information. And in Figure 15 and Figure 17, the PSCS curve changed more rapidly than the PSI curve, this phenomenon suggested that the performance of the PSCS method quickly became better with the increase of the sampling number. But when the sampling number was less than a certain number about 150, the performance of the PSCS method was also not quite good, that’s because the sparsity of those images in transform domain was about 35 as shown in Table 1, so the sampling number would preferably greater than 200 for obtaining the good performance.

As can be seen from Table 4, the accuracy of RR extracted from the smoothed respiratory waveform reconstructed by the FSCS method achieved the highest value 95.54% with acquiring 300 pressure points, and when the number of pressure points acquired was less than 300, the accuracy of RR was decreasing rapidly. However, when the system acquired more than 300 pressure points, the accuracy of RR was also decreasing at a small range instead of increasing. It indicated that the larger number of sampling points cannot evidently bring the higher accuracy of RR, this may be because the increasing of the number of sampling points for one frame led to the increasing sampling time for one frame, namely, the number of frames in a certain time would reduce. So the number of sampling points in respiratory waveform of one epoch was decreased, which resulted in the reduced performance of the waveform reconstruction. For the accuracy of RR extracted from the respiratory waveform reconstructed by the FSI method, its highest value 85.73% also occurred at 300 sampling points for one frame, but no matter how the number of pressure points changed, the accuracy of RR cannot achieve the performance as good as the FSCS method. For those experiments conducted in this study, the performance of PSCS and FSCS method were better than the PSI and FSI method. In other words, the capacity for reconstructing signals with CS method was superior to the interpolation method in almost all different number of samples retained. So we can infer that the PSCS and FSCS could perform better than PSI and FSI in similar pressure reconstruction applications.

From the relationship between the reduction of the sampling points and the reduction of the original sampling time, the sampling time was significantly decreasing with the decreasing of sampling points, namely, the hardware time consumption is rising along with the increasing of the sample number. And when the sampling number was larger than about 512, the hardware time consumption by using random sub-sampling method (the FSCS and FSI method) was more than the one by using original method, because the random sub-sampling method need the processor to generate the random measurement matrix and sent both the pressure values and the positions corresponding to those pressure values. Considering the time consumption and the accuracy of the RR, 300 points is a relatively appropriate number for sampling.

## 6. Future Work

This study mainly reduced the sampling time and increased the sampling frequency from the hardware side, but the process of the reconstruction algorithm also needs operation time. So we will compare the prime-dual interior point method used by this study with some other reconstruction algorithms such as the homotopy method, matching pursuit method and smoothed L0 method regarding to the computing speed and the reconstruction accuracy in the future.

The process of solving the least L1 norm is a course of selecting the coefficients of the signal in transform domain, and as far as we know, some classical filtering method can also be used to extract different components of original signals for getting the objective signals. So the process of the reconstructing and the filtering for the signals could be fused in one step theoretically for improving the capacity of real-time processing, and that’s one of the future work for this project.

Improve the hardware part of this system for further reducing the sampling time, such as using a FPGA and the separate circuit to replace many multiplexers used in the prototype. Improving the craftsmanship of the sensor layers and conductive layers to make the next generation of the prototype more elaborate and exquisite, meanwhile the smart mat will be made into different sizes for different groups of people with next generation.

Considering the pressure distribution image and the respiratory waveform obtained from the smart mat system, we can carry out the clinical experiments to monitor the sleep posture by the pressure distribution images and the respiratory conditions of subjects while utilizing the video and PSG recordings as reference. In order to satisfy the requirement of sleep monitoring, the improved next generation will conduct the real-time experiments in the future.

## 7. Conclusions

This study proposed a novel method to reduce the time of sampling the signals from the pressure sensor array. By utilizing this method, 40% of the sampling time can be saved by means of acquiring one-third original sampling points. The average correlation degree between reconstructed respiratory waveform and original waveform can achieve 0.9078, and the accuracy of the RR can reach 95.54% while less than one-third original sampling points were used to reconstruct the signal. And if the number of pressure points increases, the reduced time will also increase, therefore this study can open a novel way to reduce the sampling time for this smart mat system or other pressure distribution generating system.

## Figures and Tables

**Table 1:** The sparsity of pressure distribution images and number of least sampling points for recovering those images in different conditions.

Condition (the Criterion for Setting the Coefficients to 0)	Sparsity (the Number of Non-Zero Coefficients)	Number of Least Sampling Points for Reconstruction (4S)
<5% max coefficient	23.1	92.4
<4% max coefficient	29.7	118.8
<3% max coefficient	38.6	154.4
<2% max coefficient	58.3	233.2
<1% max coefficient	134.1	536.4

**Table 2:** The error rate of images reconstructed by the PSI and the PSCS method when the number of the randomly sampled points ranged from 100 to 500.

Sampled Point	AERI* (Mean% ± SD%)	
	PSI	PSCS
100	46.77 ± 3.41	39.26 ± 4.23
150	42.25 ± 3.09	31.86 ± 4.39
200	39.03 ± 2.95	24.47 ± 4.44
250	36.54 ± 2.74	18.99 ± 4.77
300	34.38 ± 2.68	15.66 ± 4.43
350	32.41 ± 2.62	13.65 ± 3.90
400	30.55 ± 2.59	12.17 ± 3.46
450	28.53 ± 2.46	10.95 ± 3.04
500	26.63 ± 2.53	9.94 ± 2.64

*AERI: Average error rate of images.

**Table 3:** The average correlation between the waves reconstructed from the PSI method with the original waves, and the average correlation between the waves reconstructed from the PSCS method with the original waves with different number of sampling points for 60 30-second epochs.

Sampled Point	Average Correlation (Mean±SD)	
	PSI	PSCS
100	0.4763 ± 0.1970	0.5419 ± 0.1956
150	0.5926 ± 0.2033	0.6558 ± 0.1968
200	0.6391 ± 0.2381	0.7662 ± 0.1544
250	0.6850 ± 0.1729	0.8473 ± 0.0899
300	0.7601 ± 0.1618	0.9078 ± 0.0783
350	0.7987 ± 0.1371	0.9332 ± 0.0545
400	0.8230 ± 0.1570	0.9486 ± 0.0533
450	0.8425 ± 0.1208	0.9623 ± 0.0278
500	0.8615 ± 0.1189	0.9704 ± 0.0309

**Table 4:** The accuracy of the RR of the respiratory waveform reconstructed by the FSI and FSCS method via five different sampling number.

Sampled Point	Accuracy of RR (Mean% ± SD%)	
	FSI	FSCS
100	81.96 ±15.68	83.51 ±12.57
200	74.13 ±14.21	84.16 ±12.61
300	85.73 ±11.86	95.54 ±5.20
400	81.05 ±9.20	91.25 ±7.64
500	77.09 ±9.92	93.33 ±5.58
